# A Systematic Review of Adjuvant Chemotherapy in Localized Dedifferentiated Chondrosarcoma

**DOI:** 10.3390/curroncol31010040

**Published:** 2024-01-19

**Authors:** Shinji Tsukamoto, Andreas F. Mavrogenis, Yuji Nitta, Alberto Righi, Tomoya Masunaga, Kanya Honoki, Hiromasa Fujii, Akira Kido, Yuu Tanaka, Yasuhito Tanaka, Costantino Errani

**Affiliations:** 1 Department of Orthopaedic Surgery, Nara Medical University, 840, Shijo-cho, Kashihara 634-8521, Japan; masunaga.t8111@gmail.com (T.M.); kahonoki@naramed-u.ac.jp (K.H.); hiromasa@naramed-u.ac.jp (H.F.); yatanaka@naramed-u.ac.jp (Y.T.); 2 First Department of Orthopaedics, School of Medicine, National and Kapodistrian University of Athens, 41 Ventouri Street, Holargos, 15562 Athens, Greece; afm@otenet.gr; 3 Department of Diagnostic Pathology, Nara Medical University, 840, Shijo-cho, Kashihara 634-8521, Japan; k140038@naramed-u.ac.jp; 4 Department of Pathology, Istituto di Ricovero e Cura a Carattere Scientifico (IRCCS) Rizzoli Orthopaedic Institute, Via di Barbiano 1/10, 40136 Bologna, Italy; alberto.righi@ior.it; 5 Department of Rehabilitation Medicine, Nara Medical University, 840, Shijo-cho, Kashihara 634-8521, Japan; akirakid@naramed-u.ac.jp; 6 Department of Rehabilitation Medicine, Wakayama Professional University of Rehabilitation, 3-1, Minamoto-cho, Wakayama 640-8222, Japan; tanakayuu717@gmail.com; 7 Department of Orthopaedic Oncology, Istituto di Ricovero e Cura a Carattere Scientifico (IRCCS) Rizzoli Orthopaedic Institute, Via Pupilli 1, 40136 Bologna, Italy; costantino.errani@ior.it

**Keywords:** dedifferentiated, chondrosarcoma, adjuvant, chemotherapy, surgery, prognosis

## Abstract

Dedifferentiated chondrosarcoma (DDCS) is a high-grade subtype of chondrosarcoma with the bimorphic histological appearance of a conventional chondrosarcoma component with abrupt transition to a high-grade, non-cartilaginous sarcoma. DDCS can be radiographically divided into central and peripheral types. Wide resection is currently the main therapeutic option for localized DDCS. Moreover, the effectiveness of adjuvant chemotherapy remains controversial. Therefore, we performed a systematic review of available evidence to evaluate the effect of adjuvant chemotherapy on localized DDCS. The purpose was to compare the 5-year survival rate among patients treated with surgery plus adjuvant chemotherapy or surgery alone for localized DDCS. The search was conducted in PubMed, Embase, and Cochrane Central Register of Controlled Trials (CENTRAL) databases. Of the 217 studies shortlisted, 11 retrospective non-randomized studies (comprising 556 patients with localized DDCS) were selected. The 5-year survival rates were similar between the two treatment groups (28.2% (51/181) vs. 24.0% (90/375), respectively). The overall pooled odds ratio was 1.25 (95% confidence interval: 0.80–1.94; *p* = 0.324), and heterogeneity I^2^ was 2%. However, when limited to peripheral DDCS, adjuvant chemotherapy was associated with prolonged survival (*p* = 0.03). Due to the paucity of included studies and the absence of prospective comparative studies, no conclusions can be drawn regarding the effectiveness or ineffectiveness of adjuvant chemotherapy for localized DDCS.

## 1. Introduction

Dedifferentiated chondrosarcoma (DDCS) is a high-grade subtype of chondrosarcoma with the bimorphic histological appearance of a conventional chondrosarcoma component with abrupt transition to a high-grade, non-cartilaginous sarcoma [1]. DDCS is responsible for approximately 2% of primary malignant bone tumors and 6–10% of chondrosarcomas [2,3]. It includes two radiographic subtypes according to the tumor location, namely central (arising from an intramedullary endochondroma) and peripheral (arising from osteochondromas of the bone cortex) [4,5,6]. Central and peripheral DDCSs are genetically distinct [7]. Dedifferentiation occurs in approximately 15% of central chondrosarcomas [8] and in approximately 6% of peripheral chondrosarcomas [9]. The average age of patients with central DDCS is 59 years, and the malignancy is slightly more common in males [8]. Patients with peripheral DDCS are slightly younger than those with central DDCS (average: 46 years) [9]. Approximately 13–56% of patients with DDCS have pathological fractures [4,8,10,11,12,13,14]. DDCS is also associated with a high risk of lung metastases (5-year survival rate: 10–24%) [8,10,15,16,17]. 

**Figure 1:**
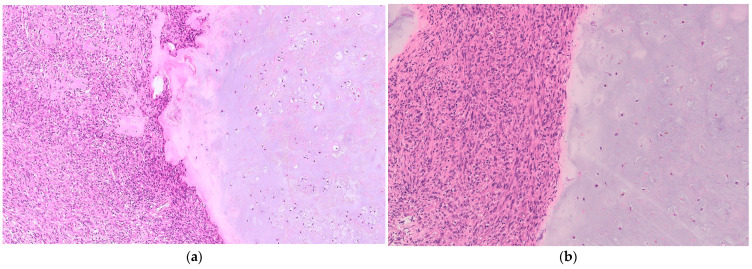
(**a**) The tumor consisted of low-grade chondrosarcoma and high-grade spindle cell sarcoma with sharp demarcation. In the low-grade chondrosarcoma component, some binucleated cells were observed (magnification: 200×). (**b**) In the high-grade sarcoma component, bizarre spindle cells were arranged in a fascicular or storiform growth pattern (magnification: 200×).

Histologically, there is an abrupt transition between the conventional hyaline cartilage and high-grade sarcoma components of DDCS [18] (Figure 1). 

The cartilaginous portion ranges from enchondroma-like appearance to grade 1–2 chondrosarcomas. High-grade dedifferentiated components exhibit characteristics of osteosarcoma or undifferentiated pleomorphic sarcoma (UPS). They rarely show features of hemangiosarcoma, leiomyosarcoma, or rhabdomyosarcoma [19]. The ratio of conventional to dedifferentiated components varies widely, and the median percentage of the dedifferentiated component is 60% (range: 2–98%) [8]. Central and peripheral DDCSs exhibit identical tumor suppressor p53 (TP53) and isocitrate dehydrogenase 1/2 (IDH1/2) mutations and share a common developmental origin [20,21]. The vast majority of DDCS cases (87%) carry IDH1/2 mutations [21]. According to a study conducted by Lucas et al., IDH mutations contributed to the early transformation of low-grade conventional chondrosarcoma to high-grade DDCS, and loss of heterozygosity at TP53 contributed to late transformation [22].

Wide resection is currently the standard treatment for localized DDCS. Nevertheless, studies suggested that perioperative chemotherapy may improve outcomes [9,11,15,23], while others reported no effect [4,8,10,12,14,24,25,26,27,28,29,30]. The estimated 5-year overall survival rate in a prospective single-arm study of 57 patients with DDCS who underwent surgery plus (neo-)adjuvant chemotherapy (surgery + NAC; methotrexate + doxorubicin + cisplatin and ifosfamide) was 39% [31]. This rate was higher than that recorded in previous retrospective analyses (range: 10–24%) [8,10,15,16,17]. According to the National Comprehensive Cancer Network guidelines, the treatment regimen used for osteosarcoma (NAC plus wide resection) should be utilized for the treatment of localized DDCS [32]. Based on the European Society For Medical Oncology guidelines, NAC can also be considered for localized DDCS [33]. However, DDCS is rare; consequently, only retrospective studies are available, while one prospective study lacks a control group [31]. There are no randomized controlled trials (RCTs) examining the efficacy of NAC against localized DDCS. Hence, the effectiveness of NAC for localized DDCS is poorly understood. Thus, we conducted a systematic review of evidence to compare the 5-year survival rate among patients treated with surgery + NAC or surgery alone (SA) for localized DDCS.

## 2. Materials and Methods

This study was performed according to the Preferred Reporting Items for Systematic Reviews and Meta-analyses 2020 statement [34]. The study protocol was registered in the UMIN Clinical Trials Registration (registration number: UMIN000052763).

### 2.1. Eligibility Criteria

The inclusion criteria were studies including human subjects; studies reporting 5-year survival after surgery + NAC or SA for localized DDCS without the detection of distant metastasis at the time of diagnosis; and literature published in English or Japanese without restriction on the year of publication. 

The exclusion criteria were animal studies; patients with distant metastases at the time of DDCS diagnosis; studies without data on 5-year survival rates or without a control group.

Patients who underwent SA for primary DDCS and were treated with palliative chemotherapy for distant metastases that developed during the disease were classified into the SA group.

### 2.2. Literature Search and Study Selection

On 26 October 2023, we performed a systematic search in PubMed, Embase, and the Cochrane Central Register of Controlled Trials (CENTRAL) databases (Table S1). Furthermore, the reference lists of the selected publications were searched for additional studies.

### 2.3. Data Collection and Presentation

The selection of studies and extraction of data were carried out independently by two investigators (S.T. and T.M.). Any disagreements were resolved through discussion between the two researchers or consultation with a third investigator. The extracted data included (1) basic data (author, year of publication, journal title, study type, study duration, follow-up period after diagnosis of DDCS, and number of patients with localized DDCS); (2) number of patients treated with surgery + NAC and SA for localized DDCS, including 5-year survival rates; (3) ratio of male-to-female patients, age, tumor location, tumor size, radiographic subtype (central or peripheral), pathological fracture, surgical margin, adjuvant radiotherapy, percentage of dedifferentiated areas in the surgery + NAC and SA groups; (4) chemotherapy regimen; and (5) histological evidence indicating necrosis after preoperative chemotherapy.

### 2.4. Data Summary, Synthesis, and Meta-Analysis

Table 1 and Table 2 provide a summary of the extracted data. The dataset included the name of the first author, year of publication, and the number of patients treated with surgery + NAC and SA for localized DDCS, including 5-year survival rates. For the comparison of 5-year survival between the surgery + NAC and SA groups, a random effects model was employed to estimate the odds ratios. We also evaluated the degree of heterogeneity between studies through inconsistency statistic (I^2^). The assessment of publications was conducted using funnel plots and Egger’s test [35]. Statistical analyses were carried out using a two-sided test (level of significance: 5%) through the ProMeta software, version 3 (INTERNOVI di Scarpellini Daniele s.a.s., Cesena, Italy) [36].

**Table 1:** Overall study characteristics.

Author [Ref. No.]	Year	Study Type	Study Period	Follow-Up Period (Years)	Patients with Localized DDCS (N)	Surgery + NAC Group (N)	Surgery + NAC Group: 5-Year Survival (N)	SA Group (N)	SA Group: 5-Year Survival (N)
Bui et al. [28]	2023	MR	2004–2022	Median: 1	47	12	3	35	14
Davies et al. [37]	2014	SR	NR	NR	2	1	0	1	0
Dickey et al. [25]	2004	SR	1986–2000	Min.: 2	37	22	1	15	2
Frassica et al. [12]	1986	SR	1915–1983	Min.: 2	50	9	2	41	4
Grimer et al. [10]	2007	MR	1975–2005	NR	242	76	25	166	42
Johnson et al. [38]	1986	MR	1948–1985	Mean: 1.3	15	2	0	13	2
Kozawa et al. [27]	2022	MR	1990–2014	Mean: 2.3	40	14	4	26	7
Liu et al. [24]	2017	SR	2008–2015	Mean: 1.2	14	5	0	9	2
Mitchell et al. [11]	2000	SR	Since 1977	Mean: 1.8	16	10	4	6	0
Staals et al. [8]	2006	SR	1969–2003	Mean: 2.8	82	24	7	58	17
Staals et al. [9]	2007	SR	1970–2002	Median: 1.2	11	6	5	5	0

DDCS, dedifferentiated chondrosarcoma; Min., minimum; MR, multi-institutional non-randomized retrospective study; NAC, (neo-)adjuvant chemotherapy; NR, not reported; SA, surgery alone; SR, single institutional non-randomized retrospective study.

**Table 2:** Overall patient characteristics.

Author [Ref. No.]	Male (%)	Mean Age (Years)	Tumors Located in the Trunk (%)	Mean Tumor Size (cm)	Radiographic Subtype	PathologiCal Fracture (%)	Patients with R0 Surgical Margin (%)	Patients Who Received Adjuvant RT	Mean Dedifferentiated Area (%)	CTX Regimen	Histologic Response Assessment: Preoperative CTX
Bui et al. [28]	NR	NR	NR	NR	NR	NR	NR	NR	NR	MAP, IFO	NR
Davies et al. [37]	0% vs. 100%	47 vs. 43	100% vs. 100%	NR	NR	NR	0% vs. 0%	0% vs. 0%	NR	NR	NR
Dickey et al. [25]	60% vs. 55%	60 vs. 69	NR	9.6 vs. 10.3	NR	NR	86% vs. 100%	0% vs. 0%	NR	MAP, IFO	NR
Frassica et al. [12]	NR	NR	NR	NR	NR	NR	NR	NR	NR	NR	NR
Grimer et al. [10]	NR	NR	NR	NR	NR	NR	NR	NR	NR	AP, AI	≥90% necrosis: 15%
Johnson et al. [38]	50% vs. 69%	54 vs. 56	50% vs. 54%	NR	NR	NR	100% vs. 69%	0% vs. 15%	NR	CYC, DOX, VCR, DTIC, CDDP, MTX	NR
Kozawa et al. [27]	NR	NR	NR	NR	NR	NR	100% vs. 100%	NR	NR	NR	NR
Liu et al. [24]	40% vs. 56%	52 vs. 46	60% vs. 56%	NR	NR	NR	80% vs. 78%	NR	NR	CDDP, EPR, IFO	NR
Mitchell et al. [11]	70% vs. 67%	47 vs. 75	40% vs. 17%	NR	Peripheral: 30%, Central: 70% vs. Peripheral: 25%, Central: 75%	10% vs. 17%	100% vs. 100%	0% vs. 0%	NR	AP, VCR	≥90% necrosis: 20%
Staals et al. [8]	NR	*	NR	n.s.	All central	NR	n.s.	NR	n.s.	MAP, IFO	≥90% necrosis: 0%
Staals et al. [9]	NR	n.s.	n.s.	n.s.	All peripheral	NR	NR	NR	52% vs. 76%	MAP, IFO	NR

Data are presented as surgery+(neo-)adjuvant chemotherapy versus surgery alone groups. AI, doxorubicin+ifosfamide; AP, doxorubicin+cisplatin; CDDP, cisplatin; CTX, chemotherapy; CYC, cyclophosphamide; DOX, doxorubicin; DTIC, dacarbazine; EPR, epirubicin; IFO, ifosfamide; MAP, methotrexate+doxorubicin+cisplatin; NR, not reported; MTX, methotrexate; RT, radiotherapy; n.s., no significant difference; VCR, vincristine. * indicates significantly younger age in the surgery+(neo-) adjuvant chemotherapy group.

### 2.5. Assessment of Methodological Quality

The quality of selected studies was independently assessed by two investigators (S.T. and T.M.). Any disagreement was resolved through discussion between the two researchers or in consultation with a third investigator. The quality of studies included in the final analysis was separately assessed using the Risk of Bias Assessment tool for Non-randomized Studies (RoBANS tool) [39].

### 2.6. Search Results

**Figure 2:**
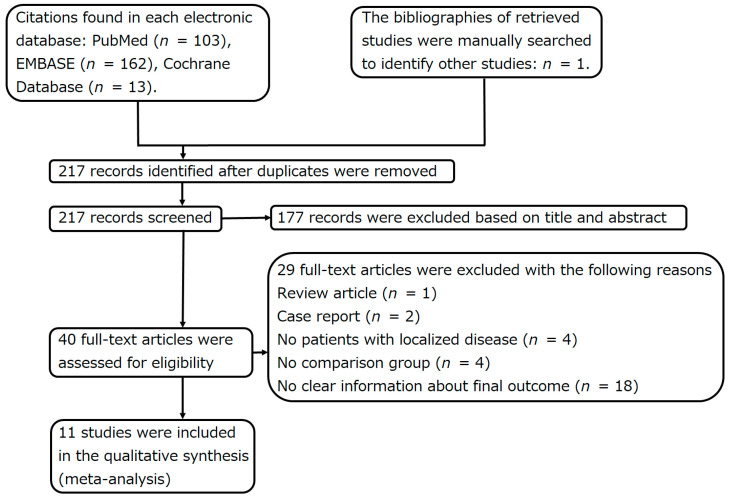
Flowchart showing the search for relevant articles.

**Figure 3:**
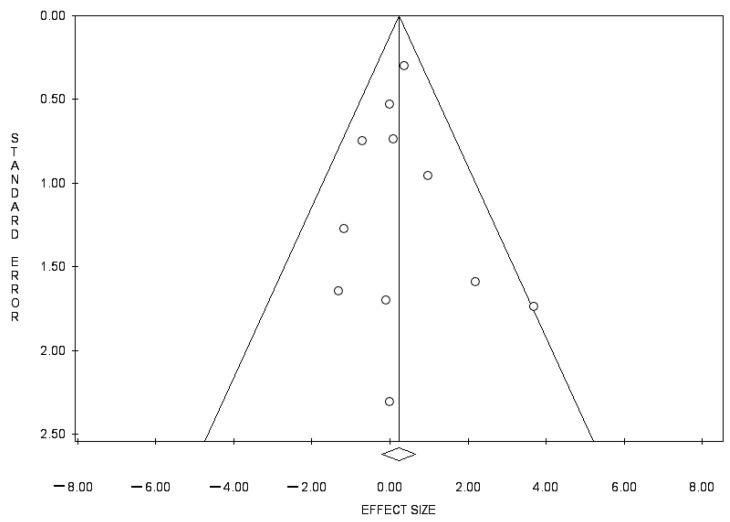
The funnel plot showing the detection of publication bias. “o” indicates each study included the Meta-Analysis.

In total, 217 studies were retrieved, and 11 of those were included in the present study (Figure 2, Table 1 and Table 2) [8,9,10,11,12,24,25,27,28,37,38]. These 11 studies were not RCTs. The results of the funnel plot of odds ratios for 5-year survival were symmetrical (Figure 3). Egger’s test (*p* = 0.880) did not indicate publication bias.

### 2.7. Demographic Data and Proportion of Patients Treated with Surgery + NAC or SA

The studies included in this analysis involved 556 patients with localized DDCS: 181 (33%) received surgery + NAC and 375 (67%) underwent SA (Table 1).

### 2.8. Methodological Quality of Included Studies

The assessment of study quality was conducted using the RoBANS tool, revealing a moderate risk of bias. In all 11 studies, the risk was as follows: “selection of participants”, high; “confounding variables”, high; “measurement of exposure”, low; “blinding of outcome”, low; “incomplete outcome data”, low; and “selective outcome reporting “, low.

## 3. Results

**Figure 4:**
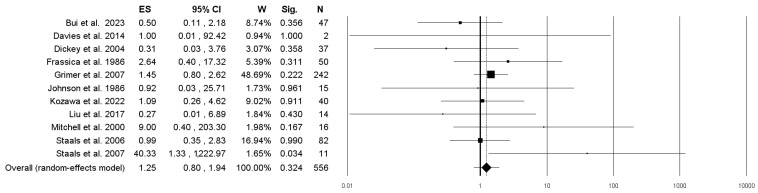
Forest plot showing the proportion of patients in the surgery combined with (neo-)adjuvant chemotherapy and surgery alone groups who survived for 5 years in different studies. CI, confidence interval; ES, effect size (odds ratio); N, total sample size; Sig., significance (*p*-value); W, weight [8,9,10,11,12,24,25,27,28,37,38].

In patients with localized DDCS without distant metastases at diagnosis, the 5-year survival rates were similar between the surgery + NAC and SA groups, i.e., 28.2% (51/181) and 24.0% (90/375), respectively. The overall pooled odds ratio was 1.25 (95% confidence interval (95%CI): 0.80–1.94; *p* = 0.324), and the heterogeneity (I^2^) was 2% (Figure 4, Table 1).

Male patients accounted for 0–70% and 55–100% of cases in the surgery + NAC group and SA group, respectively [11,24,25,37,38]. The average age ranged from 47 to 60 years and 43 to 75 years, respectively [11,24,25,37,38]. The tumor was located in the trunk in 40–100% and 17–100% of cases, respectively [11,24,37,38]. The mean tumor size was 9.6 cm and 10.3 cm, respectively [25]. The percentage of periphery DDCS was 30% and 25%, respectively [11]. Only two studies reported outcomes based on the radiographic subtype (central or peripheral) [8,9]. The risk of pathological fractures was 10% and 17% in the surgery + NAC group and SA group, respectively [11]. The rates of R0 surgical margins were 0–100% in both groups [11,24,25,27,37,38]. The proportion of patients receiving adjuvant radiotherapy was 0% and 0–15%, respectively [11,25,37,38]. The percentage of dedifferentiated areas was 52% and 76%, respectively [9]. Chemotherapy regimens included methotrexate, doxorubicin, cisplatin, ifosfamide, vincristine, epirubicin, dacarbazine, and cyclophosphamide [8,9,10,11,24,25,28,38]. The percentage of patients with ≥90% necrosis of the entire specimen in the histologic response to preoperative chemotherapy ranged from 0 to 20% [8,10,11] (Table 2).

Next, we limited the eligibility criteria to studies that distinguish between central or peripheral DDCS [8,9]. NAC was not associated with prolonged survival in localized central DDCS (*p* = 0.88) (Table 1) [8]. On the other hand, NAC was associated with prolonged survival in localized peripheral DDCS (*p* = 0.03) (Table 1) [9].

## 4. Discussion

In this study, we extracted and analyzed data comparing 5-year survival rates in patients with localized DDCS who underwent surgery + NAC versus SA. A systematic review investigating the effect of NAC on localized DDCS has not been performed thus far. The results revealed no difference in the 5-year survival rate between patients treated with surgery + NAC and those treated with SA. However, there is only one study that investigated the effect of NAC on only peripheral DDCS, and according to that study, NAC was associated with prolonged survival in localized peripheral DDCS [9]. Because there were only 11 retrospective studies and no prospective comparative studies, no conclusions can be drawn regarding the effectiveness or ineffectiveness of NAC for localized DDCS.

Kawaguchi et al. conducted a retrospective analysis of 34 patients with localized DDCS for whom treatment with ifosfamide-based chemotherapy significantly improved survival (hazard ratio: 0.2; 95%CI: 0.09–0.6; *p* = 0.003) [23]. Miao et al. also analyzed 72 patients with DDCS; they reported improved survival in patients who received chemotherapy versus those who were not treated with chemotherapy (hazard ratio: 0.23; 95%CI: 0.12–0.44; *p* < 0.001) [15]. In contrast, some studies demonstrated that NAC did not offer a benefit in patients with localized DDCS. Mercuri et al. showed that MAC did not lead to improvement in survival among 74 patients with DDCS [4]. Grimer et al. reported that NAC was not linked to better survival among 242 patients with localized DDCS (hazard ratio: 1.32; 95%CI: 0.98–1.83; *p* = 0.07) [10]. According to Nemecek et al., chemotherapy was not associated with improved survival in 33 patients with DDCS (*p* = 0.79) [14]. Furthermore, Sambri et al. did not report an association between chemotherapy and improved survival among 175 patients with DDCS of the extremities (*p* = 0.543) [26]. Gonzalez et al. carried out a study using data from the Surveillance, Epidemiology, and End Results (SEER) database. They demonstrated that, in 154 patients with localized DDCS, survival was not significantly different between the surgery and NAC group and SA group (*p* = 0.1069) [30]. Using the SEER database, Cranmer et al. concluded that, in 185 patients with DDCS, NAC did not improve survival (hazard ratio: 0.75; 95%CI: 0.49–1.12; *p* = 0.16) [29].

In the present systematic review, the percentage of patients with 90% necrosis of the entire specimen in the histological efficacy assessment of NAC ranged from 0 to 20% [8,10,11]. In previous investigations, this percentage ranged from 21 to 33% [31,40]. In conventional osteosarcoma, this percentage was 46% [41]; of note, the rate in DDCs was lower.

Many studies have evaluated prognostic factors for survival in patients with DDCS. Male sex [30], older age [10,26,30], trunk tumor location [10], larger tumor size [15,16], extraosseous extension [15], pathological fractures [10,15], metastasis at diagnosis [8,15,16,26,27,28,30], positive surgical margins [10], poor performance status [42], the use of radiotherapy [30], dedifferentiated component histological types of UPS [8,15], a high percentage of dedifferentiated components [8], and high C-reactive protein levels [14] were identified as factors of poor prognosis for survival among patients with DDCS. Trunk localization, particularly in the pelvis, has also been linked to poor prognosis [10]. Surgery in the trunk is challenging, and wide surgical margins may not be achieved. Similarly, in cases in which the tumor is large (i.e., >8 cm) or has significantly extended beyond the bone, it is difficult to achieve a radical margin; thus, such cases are associated with a lower survival rate. Survival rates are lower among patients for whom radical surgical resection is not possible and those with positive surgical margins or who require radiotherapy. In cases in which metastasis is detected at diagnosis, radical surgical resection is often challenging; hence, the detection of metastasis at this point has been linked to a low survival rate. Pathological fractures may also result in lower survival rates due to the local seeding of tumor cells via hematoma, which complicates tumor resection with negative margins [43]. Nonetheless, Sambri et al. reported that pathological fractures do not have a significant impact on the survival rate [26]. It has been shown that patients with dedifferentiated component histology of UPS have a worse prognosis than those with osteosarcoma [8,15]. This may be due to the lower sensitivity of UPS to chemotherapy compared with osteosarcoma [33,44]. The presence of a larger dedifferentiated component appears to decrease survival due to the risk of micrometastases that might be undetectable in standard staging studies at presentation [8]. However, Dehner et al. did not observe a difference in survival between patients with smaller and larger dedifferentiated components [45]. Systemic inflammation is involved in the development, progression, and metastasis of malignant tumors [46]. Therefore, the presence of high C-reactive protein levels (a marker of systemic inflammation) in patients may be correlated with poor prognosis [14].

Grimer et al. reported that many patients were unexpectedly diagnosed with DDCSs after the curettage or marginal resection of what was thought to be atypical cartilaginous tumors [10]. Positive surgical margins were associated with poor prognosis [10]. The early diagnosis of DDCS before treatment is important to improve patient prognosis [10]. The dedifferentiation rate of atypical cartilaginous tumors has been reported to be 4–6% [47,48]. Studies have reported medium-term follow-up safety for atypical cartilaginous tumors of the long bone with active surveillance [49,50,51,52]. For the active surveillance of atypical cartilaginous tumors of the long bone, magnetic resonance imaging (MRI) is recommended every 1 to 2 years [49,50,51,52,53,54,55]. A systematic review found that compared to atypical cartilaginous tumors, high-grade chondrosarcoma may more frequently exhibit the following MRI features: loss of entrapped fatty marrow, cortical breakthrough, and extraosseous soft tissue expansion [56]. Therefore, if the above findings are observed on follow-up MRI, biopsy should be considered before surgery.

Conventional chemotherapy, including doxorubicin, does not improve survival in patients with DDCS [4,8,10,12,14,24,25,26,27,28,29,30]. In recent years, research has been conducted on the mechanisms of DDCS development and therapeutic targets. The BCL2 and TGFβ have been investigated as potential gene targets in DDCS [57]. Van Oosterwijk et al. used a microarray containing 42 dedifferentiated chondrosarcomas and performed immunohistochemistry to study the expression of growth plate signaling molecules. High expression of SOX-9 and FGFR-3 was observed, along with the abnormal cellular localization of heparan sulfate proteoglycans [57]. TGFβ signaling through p-SMAD2 and PAI-1 was highly active, suggesting that TGFβ inhibitors may be a therapeutic option for DDCS [57]. Anti-apoptotic proteins (Bcl-2 and/or Bcl-xL) were also highly expressed in DDCS. Using an inhibitor with the BH-3 mimetic ABT-737 rendered the dedifferentiated chondrosarcoma cell lines sensitive to doxorubicin or cisplatin [57].

TP53 gene mutations are found in 20% of conventional chondrosarcomas and DDCSs [58]. Studies have found a correlation between the overexpression of TP53 or its point mutations and tumors with a higher histological grade. This suggests a role for this gene in tumor progression [59]. Other frequently mutated genes in chondrosarcomas are related to the cell cycle process and control, including MDM2 and cyclin-dependent kinase 4 (CDK4), which inhibit p53 and are overexpressed in chondrosarcomas [60]. The high expression of CDK4 and MDM2 correlated with a higher histological grade [61]. MDM2 overexpression has also been observed in DDCS [20]. The second most important pathway alteration involved in high-grade chondrosarcoma is in the retinoblastoma protein (pRB) pathway [61]. The deletion of CDKN2A/p16/INK4A, caused by the deletion of the 9p21 region, occurs more frequently in high-grade chondrosarcomas and DDCSs [61,62]. This suggests the potential efficacy of CDK4 inhibitors [61].

Amplification of the c-MYC oncogene is present in approximately 20% of DDCSs and correlates with poor prognosis, and the molecular targeting of MYC expression may be useful in DDCS [63]. The heterozygous loss and homozygous deletion of exostosin 1/2 (EXT1/EXT2) genes have been reported in peripheral chondrosarcomas [58,64,65]. EXT mutations are also found in osteochondromas and are much more frequent than in peripheral chondrosarcomas, suggesting an EXT-independent pathogenesis of secondary peripheral chondrosarcomas [66].

Genomic profiling has revealed telomerase reverse transcriptase (TERT) gene amplification and ATRX mutations, in addition to TERT promoter mutations, in approximately 20% of high-grade chondrosarcomas and DDCSs. These telomere gene abnormalities are accompanied by IDH1/IDH2 mutations, CDKN2A/2B deletions, and TP53 mutations, suggesting a possible association and synergistic effect between these genes in chondrosarcoma progression [67]. Therefore, treatments targeting telomerase may be effective for treating DDCS [68].

Below are reports of a new drug administered to patients with DDCS. The administration of sirolimus (mTOR inhibitor) and cyclophosphamide to a patient with DDCS resulted in a progression-free survival of 26.7 months [69]. GDC-0499 (Hedgehog pathway inhibitor) was administered to five patients with DDCS; however, progressive disease was observed in all patients [70]. Bupathi et al. treated a patient with DDCS using pazopanib (a multi-targeted tyrosine kinase inhibitor of vascular endothelial growth factor receptor (VEGFR), platelet-derived growth factor receptor (PDGFR), and c-Kit); stable disease was observed in those patients [71]. Ivosidenib (a selective inhibitor of mutant IDH1) was administered to six patients with DDCS; only 30% did not experience disease progression at 3 months, while all patients had disease progression at 6 months [72].

Increasing clinical evidence indicates that immunotherapy might be effective against advanced DDCS [73,74,75,76,77]. Kostine et al. analyzed DDCS tissues, showing the expression of programmed cell death 1- ligand 1 (PD-L1) in 52% of samples and large T-cell infiltration [73]. Iseulys et al. analyzed a sample of 49 patients with DDCS and found that 43% of them exhibited positivity for PD-L1 [74]. The investigators showed that tumor-associated macrophages are the predominant type of immune cells in the immune environment of chondrosarcoma and that anti-PD-L1 therapy is indicated for DDCS [74]. Paouzzi et al. administered nivolumab (anti-PD-1 antibody) to two patients with DDCS: one with stable disease and one with partial response [75]. In a phase II trial evaluating pembrolizumab (anti-PD-1 antibody) in bone sarcoma, 20% of patients with DDCS had a partial response [76]. Singh et al. treated a patient with PD-L1-positive DDCS using pembrolizumab; the patient exhibited a durable complete response for 24 months [77].

The limitation of this study should be acknowledged. The analysis included only retrospective studies with an indication bias toward NAC. However, there were no significant differences between the two groups in the proportion of male patients, age, tumor location, size, peripheral versus central location, percentage of patients with pathological fractures, percentage of R0 surgical margin, percentage of patients receiving adjuvant radiotherapy, and percentage of dedifferentiated areas (Table 2). The random allocation of participants into groups in RCTs avoids several of these biases. Considering the lack of RCTs in this field, well-designed cohort and observational studies with strong effects might yield important findings.

## 5. Conclusions

Due to the paucity of included studies and the absence of prospective comparative studies, no conclusions can be drawn regarding the effectiveness or ineffectiveness of NAC for localized DDCS. 

## Supplementary Materials

The following supporting information can be downloaded at: https://www.mdpi.com/article/10.3390/curroncol31010040/s1, Table S1: Search strategy.
