# Supplementary material for: A Systematic Review of Adjuvant Chemotherapy in Localized Dedifferentiated Chondrosarcoma
Source: Curr Oncol. 2024 Jan 19;31(1):566–78. doi: 10.3390/curroncol31010040 (PMC10813944; doi:10.3390/curroncol31010040)
Supplement: Supplementary file 1 [file curroncol-31-00040-s001.zip › curroncol-2777539-supplementary.pdf]

Supplementary Material

Table S1: Search strategy

	"dedifferentiated chondrosarcoma" "chemotherapy"	2023/10/26
PubMed		
1	dedifferentiat*[tiab] AND (chondrosarcoma*[tiab] OR "Chondrosarcoma"[mh])	527
2	"Drug Therapy"[mh] OR drug therapy[sh] OR drug thepar*[tiab] OR chemotherap*[tiab] OR pharmachotherap*[tiab]	3677217
3	#1 AND #2	103
Embase		
1	SU("dedifferentiated chondrosarcoma") OR TI,AB(dedifferentiat* AND chondrosarcoma*)	673
2	SU("drug therapy") OR TI,AB("drug therap*" OR chemotherap* OR pharmachotherap*)	6095442
3	S1 AND S2	162
Cochrane		
CENTRAL		
1	dedifferentiat* AND chondrosarcoma*	13
